# The effectiveness of workplace interventions to increase physical activity and decrease sedentary behaviour in adults: protocol for a systematic review

**DOI:** 10.1186/s13643-015-0166-4

**Published:** 2015-12-12

**Authors:** Christina C. Loitz, Robert J. Potter, Jessica L. Walker, Nicole C. McLeod, Nora J. Johnston

**Affiliations:** Alberta Centre for Active Living, Faculty of Physical Education and Recreation, University of Alberta, 3rd floor, 11759 Groat Road, T5M 3K6 Edmonton, Alberta Canada; UWALK, Faculty of Physical Education and Recreation, University of Alberta, 3rd floor, 11759 Groat Road, T5M 3K6 Edmonton, Alberta Canada

**Keywords:** Physical activity, Sedentary behaviour, Adults, Workplace, Occupational, Systematic review, Knowledge mobilization

## Abstract

**Background:**

A physically active lifestyle plays a preventative role in the development of various chronic diseases and mental health conditions. Unfortunately, few adults achieve the minimum amount of physical activity and spend excessive time sitting. Developing targeted interventions to increase active living among adults is an important endeavour for public health. One plausible context to reach adults is the workplace. This systematic review aims to review the effectiveness of workplace interventions on increasing physical activity and decreasing sedentary behaviour in the workplace.

**Methods:**

An advisory group of practitioners will work in collaboration with the research team to inform the systematic review and knowledge mobilization. Fifteen electronic databases will be searched to identify studies examining the effectiveness of workplace interventions on physical activity and sedentary behaviour. All experimental designs and observational studies (non-experimental intervention studies) meeting the study criteria will be included. Studies examining generally healthy, employed, adult participants will be included for the review. Interventions will focus on increasing physical activity and/or decreasing sedentary behaviour from the individual to policy level. The primary outcome variables will be reported or observed physical activity and/or sedentary behaviour in the workplace. Secondary outcomes will include variables ranging from return on investment to quality of life. Study quality will be assessed for risk of bias following the protocol identified in the *Cochrane Handbook for Systematic Reviews of Interventions* and supplemented by the guidelines developed by the Cochrane Effective Practice and Organisation of Care group, using RevMan. The quality of the evidence will be assessed using the Grading of Recommendations, Assessment, Development and Evaluation approach. Meta-analyses, forest plots, and harvest plots will be used where appropriate to assess the direction, size, and consistency of the intervention effect across the studies using similar intervention strategies. Follow-up knowledge mobilization activities and products will be developed to support the use of this knowledge in practice.

**Discussion:**

This protocol paper describes a systematic review assessing the effectiveness of various types of workplace interventions on increasing physical activity and decreasing sedentary behaviour at work. Collaborating with an advisory group of potential knowledge users throughout the process postulates a greater use and reach of the information gained from this systematic review by knowledge users.

**Systematic review registration:**

PROSPERO CRD42015019398

**Electronic supplementary material:**

The online version of this article (doi:10.1186/s13643-015-0166-4) contains supplementary material, which is available to authorized users.

## Background

Physical inactivity and excessive sedentary behaviour are two current global public health concerns [1]. Being physically active plays a preventative role in developing cardiovascular disease, type 2 diabetes, obesity, and some forms of cancer [2]. For adults, being physically inactive refers to not achieving the physical activity guidelines of 150 minutes of moderate-to-vigorous physical activity per week [3]. Researchers have identified sedentary behaviour as an additional and distinct negative health behaviour, which is different from physical inactivity [1, 4]. Being sedentary refers to spending excessive time sitting or in a reclined posture while participating in activities with an energy expenditure ≤1.5 metabolic equivalents during wake time [5]. Currently, public health guidelines for sedentary behaviour exist for Canadian children, youth, and the early years (0 to 4 years) [6, 7]. A recent consensus statement has been published to provide employers sedentary behaviour guidelines for desk-based employees in the UK [8]. These guidelines suggest employees engage in 2 hours of daily standing or light physical activity at work which would progressively increase to 4 hours per day [8]. Surveillance studies have found that on average, people spend more than one half of their waking day in sedentary activities [9]. Studies have reported a positive relationship between sedentary behaviour and all-cause mortality, cardiovascular disease, cancer (breast, colon, colorectal, endometrial, and epithelial ovarian), and type 2 diabetes in adults [10]. Engaging in excessive sedentary behaviour time is detrimental even to those who meet current physical activity guidelines of 150 min of moderate-to-vigorous intensity physical activity per week [10, 11]. A recent meta-analysis supported the distinction between physical inactivity and sedentary behaviour as associated with various chronic diseases and mortality; however, adverse outcome effects associated with sedentary time generally decreased in magnitude among persons who participated in higher levels of physical activity compared with lower levels [10].

Data from the Canadian Fitness Lifestyle Research Institute reported that 38 % of adults had heard of the Canadian Physical Activity Guidelines (CPAG), while only 8 % of these respondents were able to correctly identify the guidelines [12]. Adults are a difficult population to reach for public health messages and interventions, as numerous factors compete for their attention. Strategic methods are required to influence hard to reach populations and observe population-level health improvements associated with the widespread adoption of evidence-based strategies [13]. A strategic method is to approach adults through the workplace. The broad reach of workplaces makes this setting an ideal location to influence adults with a range of physical activity and sedentary behaviour experiences and habits [14]. More specifically, targeting adults with sedentary jobs, such as office workers, may improve physical activity participation and reduce sedentary behaviour time at a population level [14]. Workplace interventions can take many forms including participation in competitions and challenges, education and counselling, shifting cultures and norms, and modifying workstations and the physical environment [15]. The degree of success, level of practicality, influence on productivity, and cost-effectiveness of physical activity and sedentary behaviour interventions are critical to occupational health practitioners in the development and implementation stage of an intervention. Additionally, knowledge of secondary outcomes associated with physical activity and sedentary behaviour workplace interventions are necessary for many practitioners to persuade upper management of the benefits to the employee and overall workplace.

Practitioners are looking for evidence-based programmes and policies built on the best available scientific evidence to develop and implement successful and cost-effective public health interventions [11, 13]. Although systematic reviews of academic literature were ranked the most important scientific resources for practitioners, access to journals was a common constraint to their use in public health [16]. Consultation across academia, policy, and practice may be one method of increasing the usefulness, relevance, and reach of scientific findings [16]. Consulting with prospective end users of research studies and systematic reviews, such as practitioners, in the project development, implementation, and reporting may increase the practicality and use of the knowledge gathered to a broader audience and increase positive public health outcomes.

The purpose of this project is to conduct a systematic review of the effectiveness of workplace interventions on increasing physical activity and decreasing sedentary behaviour at the workplace in consultation with a group of researchers and practitioners, as described in this protocol paper.

## Methods

### Study design

An advisory group consisting of practitioners (*n* = 9) and researchers (*n* = 3) with an interest in reducing sedentary behaviour and increasing physical activity among adults in the workplace has been formed to inform the systematic review and follow-up knowledge mobilization activities. The study questions and a draft of the search and extraction strategy will be guided by the population, intervention, comparison and outcome (PICO) method [17] and will be developed in consultation with the advisory group. The systematic review will follow the methods described in the Cochrane handbook [18] and will be reported in accordance with the PRISMA statement [19]. The Grading of Recommendations, Assessment, Development and Evaluation (GRADE) will be used to assess the quality of evidence related to the risk of bias, directness of the evidence, consistency and precision of the results, and likelihood of publication bias [20].

Knowledge mobilization activities or products will be developed following the guidance of the advisory group. These activities and products will be tailored to the needs of people working in human resources, health promotion and exercise leadership to assist with the implementation of effective health initiatives to increase physical activity and reduce sedentary behaviour in the workplace.

### Study registration

This systematic review is registered with PROSPERO (registration number: CRD42015019398; www.crd.york.ac.uk/PROSPERO).

### Criteria for considering studies for this review

#### Type of studies

Any research study (experimental or non-experimental observational studies of interventions) exploring physical activity or sedentary behaviour interventions in the workplace will be considered for this systematic review. This will include randomized trials, non-randomized trials, cluster randomized trials, controlled before-after studies, repeated measures studies, interrupted time series studies, and before-after studies as defined by Effective Practice and Organisation of Care (EPOC) [21]. Workplace interventions are often delivered at the group level. However, only studies examining the individual level effect will be included in this review. Control groups will be used for comparison when available, but no restrictions will be placed on the comparison group.

#### Types of participants

Only studies conducted in a western/developed country, with apparently healthy adults over 18 years of age working in a full- or part-time capacity, will be included in this review. Studies that focus on specific comorbidities or diseases (i.e. diabetes, arthritis, cancer, stroke), special populations (i.e. pregnant, physical disability, or cognitive disability), or targeting pain management or musculoskeletal issues will be excluded. Additionally, studies including non-employee participation (i.e. students) will be excluded, if the data were not separated. Studies will predominantly focus on sedentary administrative positions; however, to broaden the scope of this review to include both physical activity and sedentary behaviour interventions, the type of occupations will not be a criteria for exclusion.

#### Types of interventions

We will include studies utilizing various intervention strategies in isolation or in combination to increase physical activity and/or decrease sedentary behaviour. These intervention strategies will be grouped into preliminary categories and intervention activities listed in Table 1.

**Table 1:** Physical activity and sedentary behaviour workplace intervention strategy categories

Strategy categories	Exemplar intervention activities
Access and the physical environment	Introduce workstations that support physical activity or deter sedentary behaviours (e.g. sit-to-stand desks, treadmill workstations, cycling workstations, stepping workstations). Add environmental changes to the workplace to facilitate physical activity (e.g. exercise facility, bike racks in secure space).
Organizational culture and norms	Create an office environment that supports active breaks (e.g. stair use, walking meetings). Encourage active and frequent breaks to sit less (e.g. hourly prompts to stand up or walk).
Information and counselling	Provide individual or group counselling (e.g. individual or group goal setting and on-site consultation with a health and wellness expert). Provide Internet-based tools and feedback (e.g. websites, online forums, tailored messaging or feedback via email, phone applications).
Workplace challenges or competitions	Pedometer or stair climbing challenges (e.g. 10,000-step daily challenge, walk across Canada pedometer challenge, stair climbing challenges). Monitoring physical activity using log books (e.g. daily physical activity and sedentary behaviour log books, physical activity and sedentary behaviour monitors).

#### Types of comparators

This review will include various study designs with a control group or comparator group (e.g. randomized trials, controlled before-after studies, non-randomized trials), while other eligible studies will not have a comparator (e.g. repeated measures studies). No restrictions will be made on the control or comparison group studies (e.g. no or alternate types of physical activity interventions and/or sedentary behaviour interventions), as many studies occur in naturalistic workplace settings.

#### Types of outcome measures

The primary outcomes will be changes in physical activity and sedentary behaviour. Changes in physical activity will be reported in minutes of physical activity per day (moderate-to-vigorous physical activity and total physical activity), energy expenditure due to physical activity participation (MET minutes per day), and steps per day. Sedentary behaviour will be reported as changes in sedentary behaviour time (minutes per day). Articles reporting changes in physical activity or sedentary behaviour over a 7 or 5-day time period will be recalculated to daily values, such as minutes or steps per day. Physical activity and sedentary behaviour assessed using direct measures (e.g. accelerometers, pedometers) and self-report measures (e.g. questionnaires, surveys, journals, and logbooks) will be accepted. The secondary outcomes will include the various benefits associated with increased physical activity and reduced sedentary behaviour that are specifically relevant to workplaces. Some identified secondary outcomes include but are not limited to workplace absenteeism or sick days, presenteeism or work productivity, quality of life and mental or physical wellbeing, and return on investment. The measurement of these factors may vary in methods and units.

#### Search methods for the identification of studies

A comprehensive search strategy will be designed by the researchers with the assistance of a librarian to search for all eligible published and unpublished studies and dissertations written in the English language. The search strategy will be based on our target population, the work environment, and all terms related to physical activity and sedentary behaviour. Search criteria will be created alongside a librarian, in consultation with protocol created by Christie and colleagues [15] and the search terms proposed by the advisory group consisting of practitioners and researchers. We will search the following electronic databases from 2004 to present to identify potential studies: *Ovid MEDLINE In-Process & Other Non-Indexed Citations, Ovid MEDLINE(R) Daily and Ovid MEDLINE 1946 to Present* (Ovid); *EMBASE 1974-Present* (Ovid); *PsycInfo 1987-Present* (Ovid); *EBM Reviews: Cochrane Database of Systematic Reviews, SPORT Discus 1975-Present* (EBSCO); *CINAHL Plus with Full-text 1937-Present* (EBSCO); *ProQuest Dissertations & Theses International*; *ABI Inform Complete, Business Source Complete, Human Resources Abstracts, Scopus*; and *Web of Science*. Conference proceedings will be searched via *Web of Science* and *EMBASE*. Dissertations and theses will be searched via *ProQuest Dissertations & Theses Full-text* and *ProQuest Dissertations & Theses Global*. The proposed search strategy terms for Medline are listed in Table 2 and will be modified to fit the index system of other databases. The reference list of articles meeting the inclusion criteria will be scanned for additional eligible studies.

**Table 2:** Sample MEDLINE search strategy terms

Workplace terms
1. Workplace/
2. Work/
3. Employment/
4. ((company or companies or human resource* or business*) adj3 (work* or staff or personnel or culture* or organi?ation*1 or environment* or sociocultural or policy or policies or climate* or infrastructure* or design* or layout* or scheme*or program*)).ti,ab.
5. ((factory or factories or office*) adj3 (work* or staff or personnel or culture* or organi?ation*1 or environment* or sociocultural or policy or policies or climate* or infrastructure* or design* or layout* or scheme* or program*)).ti,ab.
6. (“small and medium enterpri?e*” or SMEs or SME).ti,ab.
7. (worker* or workplace* or worksite* or staff or colleagues).ti,ab.
8. (employee* or employer*).ti,ab.
9. (work place* or work site* or work location* or work setting*).ti,ab.
10. ((job* or employment) adj2 (place* or site* or location* or Setting* or personnel)).ti,ab.
11. Occupational health services/
12. 1 OR 2 OR 3 OR 4 OR 5 OR 6 OR 7 OR 8 OR 9 OR 10 OR 11
Physical Activity and Sedentary Behaviour terms
13. exp Sports/
14. (running or jogging or dance* or dancing or ballet or badminton or tennis or swim* or racquet sport* or squash or pilates or spinning class* or step* class* or yoga or yogalates or gym*1 or gymnasia or football or rugby or netball or cricket or bowling or tai chi).ti,ab.
15. (physical* adj3 (fit* or activit*3 or inactiv*3 or training or exercise* or sport*)).ti,ab.
16. (aerobics or sedentary or fitness).ti,ab.
17. (aerobic* adj3 (activit* or exercise*)).ti,ab.
18. ((climb* or us* or walk*) adj2 stair*).ti,ab.
19. “active at work”.ti,ab.
20. (shower* adj3 (provi* or access* or facilit* or availabl*)).ti,ab.
21. ((us* or tak*) adj1 (public transport* or bus*2 or train*1 or tram*)).ti,ab.
22. (walk* or bik* or cycling or bicycling or bicycle* or commut*). ti,ab.
23. ((subsid* or voucher*) adj5 (gym* or sport* or leisur* or swim* or exercis* or public transport* or bus*2 or train*1 or tram*)).ti,ab.
24. (leisure pass* or active travel*).ti,ab.
25. Sedentary
26. Sedentary Behaviour
27. Sitting
28. Screen time, computer-use, TV viewing
29. 13 OR 14 OR 15 OR 16 OR 17 OR 18 OR 19 OR 20 OR 21 OR 22 OR 23 OR 24 OR 25 OR 26 OR 27 OR 28
Combining search terms
12 AND 29

#### Selection of studies

Articles will be imported into the RefWorks (ProQuest, Ann Arbor, MI, USA) bibliographic management programme, where duplicates will be removed. Two reviewers (RJP, NCM) will independently screen all identified titles and abstracts from the systematic search to identify potentially relevant studies for inclusion according to PICO (see Table 3 for PICO-proposed criteria for inclusion in the systematic review) and label each study as an article to include or exclude, or if it is unclear. Full text articles of studies labelled ‘include’ or ‘unclear’ will then be retrieved and independently screened by two review authors (RJP, JLW). All reasons for exclusion of ineligible studies will be recorded. A third author (CCL) will be consulted to resolve any disagreements. Reviewers will not be blinded to the journal authors. The results of the selection process will be reported in detail using the Preferred Reporting Items for Systematic Reviews and Meta-Analyses (PRISMA) flow diagram [19]. A completed copy of the PRISMA checklist will be provided as part of the final reporting (Additional file 1).

**Table 3:** Population, intervention, comparison and outcome (PICO)-proposed criteria for inclusion in the systematic review

Acronym	Term	Description
P	Population	Apparently healthy, working age, adult employees (≥18 years of age) from Western/developed countries.
I	Intervention	Interventions were delivered within the workplace with a focus on increasing physical activity and/or decreasing sedentary behaviour. These interventions may include but are not limited to the following: workplace challenges or competition, education and counselling, changes to workplace culture and policy, and/or changes to the physical work environment or in access to facilities.
C	Comparison	This review will include all experimental study designs. Control groups will be used for comparison when available, but no restrictions will be placed on the comparison group.
O	Outcomes	Primary outcomes will be a change in physical activity and/or sedentary behaviour. The secondary outcomes will include various other benefits associated with the intervention and relevant to the workplace. These outcomes may include but are not limited to the following: absenteeism or sick days, presenteeism or work productivity, quality of life, mental health, and/ or return on investment.

#### Data extraction and management

Study characteristics will be independently extracted from each study following a guided data collection form for study characteristics and outcome data by RJP and JLW. Inconsistencies or disagreements will be resolved through discussion and consultation with CCL. The following characteristics will be included:

- Publication details: authors, year, and country of study
- Methods: study design, baseline measure, duration of intervention, time points when data were collected, and study setting (location, year, and environment)
- Participant characteristics: number of participants, mean age or age range, gender ratio, and participant inclusion criteria and exclusion criteria
- Interventions: description of intervention and comparison/control group and number of participants allocated to each group
- Outcomes: description of primary and secondary outcomes, list of measurement tools and devices, unit of measurement for primary and secondary outcomes, and intervention effects on the primary and secondary outcomes (effect size, 95 % CI, standard mean deviation)
- Notes: any additional information that may express conflict of interest or bias

Extracted information will be inputted into the ‘characteristics of included studies’ table described above. The first ten articles will be extracted independently by three authors (RJP, CCL, JLW) to pilot the extraction plan. Changes in physical activity and sedentary behaviour scores and standard deviation scores will be calculated according to Higgins and Green [18] when necessary and appropriate data are available. Authors, of included studies with missing or incomplete data, will be contacted by email to retrieve further information. The research team will present data from the characteristics tables to the advisory group, to assist with the classification and identification of relevant and representative intervention categories, as a broad range of workplace sedentary behaviour and physical activity interventions are expected. The intervention categories identified in Table 1 will be used as a starting point and may be adjusted and/or new categories may be created.

#### Risk of bias and quality within studies

Included randomized controlled trials will be assessed for risk of bias using the Cochrane Risk of Bias Tool; non-randomized studies, including non-randomized controlled trials, controlled before-after studies, and interrupted time series, will be assessed according to the suggested risk of bias criteria for EPOC reviews [22]. Included studies will be assessed for six types of bias (selection, performance, detection, attrition, reporting, and other bias) from seven risks of bias domains (random sequence generation, allocation concealment, blinding of participants and personnel, blinding of outcome assessment, incomplete outcome data, selective reporting, and other potential threats to validity) as reported in *Cochrane’s Handbook for Systematic Reviews of Interventions*, Section 8.4.4 [18]. The potential source of bias for each domain will be graded as high, low, or unclear in conjunction with a quote or reviewer’s comment to justify the decision. For instance, individual studies will be rated as a low risk for selection bias, if the study reports sufficient information about participant randomization, random sequence generation, and an appropriate method to conceal allocation. Conversely, studies will be graded with high risk if there is a lack of control group, and as such, no procedures to conceal allocation were in place. Special attention will be placed on assessing risk of bias for non-randomized trials as particular concerns arise with respect to differences between people in different intervention groups (selection bias). Data will be presented in both a ‘risk of bias graph’, which illustrates the proportion of studies rated as ‘low risk’, ‘high risk’, or ‘unclear risk’ of bias for each of the seven risks of bias domains, and a ‘risk of bias summary’ figure which presents all the judgements in a cross-tabulation format for each of the included studies in the review.

#### Quality of the evidence

The quality of evidence for the four proposed categories of physical activity and sedentary behaviour interventions (see Table 1) will be assessed as high, moderate, low, or very low using the GRADE approach [23]. The GRADE approach rates randomized trials and double-upgraded observational studies as the highest quality of evidence and case series and triple-downgraded randomized trials or downgraded observational studies as the lowest quality of evidence [24]. In addition to study design, the quality of evidence will be assessed based on inconsistency, imprecisions, indirectness, and publication bias. Justifications to downgrade or upgrade the quality of evidence will be supported through footnotes and comments. We will follow the methods and recommendations outlined in Sections 11 and 12 of the *Cochrane Handbook for Systematic Reviews of Interventions* [18]. The risk of bias assessed using RevMan 5.3.3 will be imported into GRADE Profiler (GRADEpro) Version 3.6 (McMaster University, Hamilton, ON, Canada) in order to rate the quality of evidence and create a summary of findings table.

#### Analyses

Separate analyses will be conducted for each intervention category listed in Table 1 for physical activity, sedentary behaviour, and combined physical activity and sedentary behaviour interventions, as workplace interventions may include a broad range of strategies. As previously mentioned, the ‘characteristics of included studies’ table will be created to identify the study design (methodology, duration), population (participant characteristics), intervention (strategies), comparison (comparators), and outcome (type and unit of measurement).

Meta-analyses, forest plots, and harvest plots will be used to assess the direction, size, and consistency of the effect across the studies. Judgements by three researchers (JLW, RJP and CCL) will determine the appropriate analyses for the studies based on the homogeneity of the studies, as well as the quality and breadth of the literature included. Both quantitative and narrative syntheses will be considered to assess the intervention effect, and the strength of the evidence will be assessed according to narrative synthesis.

If sufficient data are available, forest plots and meta-analyses will be developed using RevMan 5.3.5 to synthesize the measures of effect (e.g. mean difference) and 95 % confidence intervals for each intervention on physical activity and sedentary behaviour in each of the four proposed categories of interventions. Forest plots and meta-analyses will only be performed when the included studies are sufficiently homogeneous in terms of study design, interventions, and outcome units measured (e.g. minutes/day of physical activity) to provide a meaningful and succinct summary. It is important to note that workplace physical activity and sedentary behaviour interventions may encompass too broad of a scope within the four proposed categories of intervention strategies listed in Table 1 to permit meta-analyses.

Due to the variety of measures (e.g. energy expenditure, time, steps) and units of measurement (e.g. minutes per day or week, MET minutes per day or week, steps per day or week) for physical activity and sedentary behaviour evidence, harvest plots will also be utilized. The harvest plots will synthesize and graphically display a summary of the effect of intervention results from the studies that were not appropriate for the forest plots [24]. As such, the harvest plots will permit evidence across various exposures to be compared.

#### Subgroup analyses

In addition to the primary analyses, several subgroup analyses will be conducted if sufficient data are available. These analyses may examine differences between the following: self-reported and objectively measured physical activity and sedentary behaviour exposures; intervention categories (outlined in Table 1); intervention focus (e.g. pedometer walking challenge versus sit-to-stand workstations); intervention delivery (e.g. single versus multi-modal); study design (e.g. control group versus no control group, randomized control trial versus non-randomized trial); comparison group type (e.g. standardized practice versus modified physical activity or sedentary interventions); studies with high versus low risk of bias; and length of intervention.

#### End of project knowledge mobilization activities

Once the analyses are complete, the advisory group will be consulted to review and assist in the interpretation of the findings from the systematic review. This will be followed by the advisory team consulting on the development of knowledge mobilization activities and products. The findings from the systematic review and the previously identified knowledge mobilization goals will be reviewed and discussed to assist in the development of a knowledge mobilization plan. More specifically, the advisory group and research team will identify and consider the prospective knowledge users; the main messages from the systematic review; and appropriate methods of translating, communicating, and disseminating the knowledge mobilization product or activity according to the Knowledge Translation Planning Template^©^ [25].

### Discussion

This systematic review is the first to our knowledge to examine the effectiveness of sedentary behaviour and physical activity interventions in the workplace according to the type of intervention strategy. Classifying the target behaviour of workplace interventions as physical activity, sedentary behaviour, or both is expected to be challenging, as many physical activity interventions include a sedentary behaviour component, and vice versa. A recent systematic review found interventions in various contexts that exclusively target sedentary behaviour resulting in large and clinically meaningful reductions in sedentary behaviour, whereas interventions jointly targeting sedentary behaviour and physical activity produced less consistent findings and smaller reductions in sedentary behaviour time [26]. This highlights the importance of disentangling the intervention type as physical activity, sedentary behaviour, or both, rather than categorizing studies solely by the outcome measure.

A unique aspect of the procedures in this systematic review is the inclusion of an advisory group of end users from the start of the project planning. An advisory group of various potential end users (e.g. occupational health practitioners, health and fitness professionals, not-for-profit health promoters, and a policymaker) were included and will continue to be included in the planning process for the systematic review and follow-up knowledge mobilization activities. Members of the prospective user groups will be involved throughout the process, which includes developing the purpose statement, reviewing the protocol and findings, creating knowledge mobilization products and planning dissemination strategies. End user involvement throughout the project is hypothesized to increase uptake of findings from the systematic review into practice. This systematic review will provide a rigorous examination of the effectiveness of workplace interventions while considering the challenges and limitations associated with conducting tight experimental designs in a naturalistic workplace setting and insight from practitioners that are in a position to implement workplace programmes and policies to address physical inactivity and excessive sedentary behaviour.

## Additional file

Additional file 1:
**PRISMA-P checklist. **(DOC 83 kb)

## Abbreviations

CPAG: Canadian Physical Activity Guidelines
EPOC: Effective Practice and Organisation of Care
GRADE: Grading of Recommendations, Assessment, Development and Evaluation
PICO: Population, intervention, comparison and outcome
PRISMA: Preferred Reporting Items for Systematic Reviews and Meta-Analyses
PROSPERO: prospective register of systematic reviews
MET: metabolic equivalent of task
